# Correction to: The genome of the zebra mussel, *Dreissena polymorpha*: a resource for comparative genomics, invasion genetics, and biocontrol

**DOI:** 10.1093/g3journal/jkac272

**Published:** 2022-10-19

This is a correction to: Michael A McCartney, Benjamin Auch, Thomas Kono, Sophie Mallez, Ying Zhang, Angelico Obille, Aaron Becker, Juan E Abrahante, John Garbe, Jonathan P Badalamenti, Adam Herman, Hayley Mangelson, Ivan Liachko, Shawn Sullivan, Eli D Sone, Sergey Koren, Kevin A T Silverstein, Kenneth B Beckman, Daryl M Gohl, The genome of the zebra mussel, *Dreissena polymorpha*: a resource for comparative genomics, invasion genetics, and biocontrol, *G3 Genes|Genomes|Genetics*, Volume 12, Issue 2, February 2022, https://doi.org/10.1093/g3journal/jkab423

In the originally published version of this manuscript, an alternative version of the assembly was used to construct Figure 5 (and the associated Supplementary File 13). Figure 5 and Supplementary File 13 have been replaced with the corrected versions.

In addition, the gene IDs (locus tags) were inadvertently reassigned upon uploading the genome to NCBI. Thus, the gene IDs in the GenBank genome do not match those reported in the original publication. To minimize confusion, the gene IDs in GenBank will be used for zebra mussel genes. The gene names referenced in this publication are in the “orig_transcript_id” and “orig_protein_id” fields of the GenBank GFF file. An additional file was also attached to the publication (“mapping_table.txt”) with the correspondence between the NCBI/GenBank gene names and gene names used in this paper and associated supplemental materials.

These errors have been corrected.

