# Supplementary material for: The genome of the zebra mussel, Dreissena polymorpha: a resource for comparative genomics, invasion genetics, and biocontrol
Source: G3 (Bethesda). 2021 Dec 13;12(2):jkab423. doi: 10.1093/g3journal/jkab423 (PMC9210306; doi:10.1093/g3journal/jkab423)
Supplement: jkab423_Supplementary_Figures [file jkab423_supplementary_figures.pdf]

a

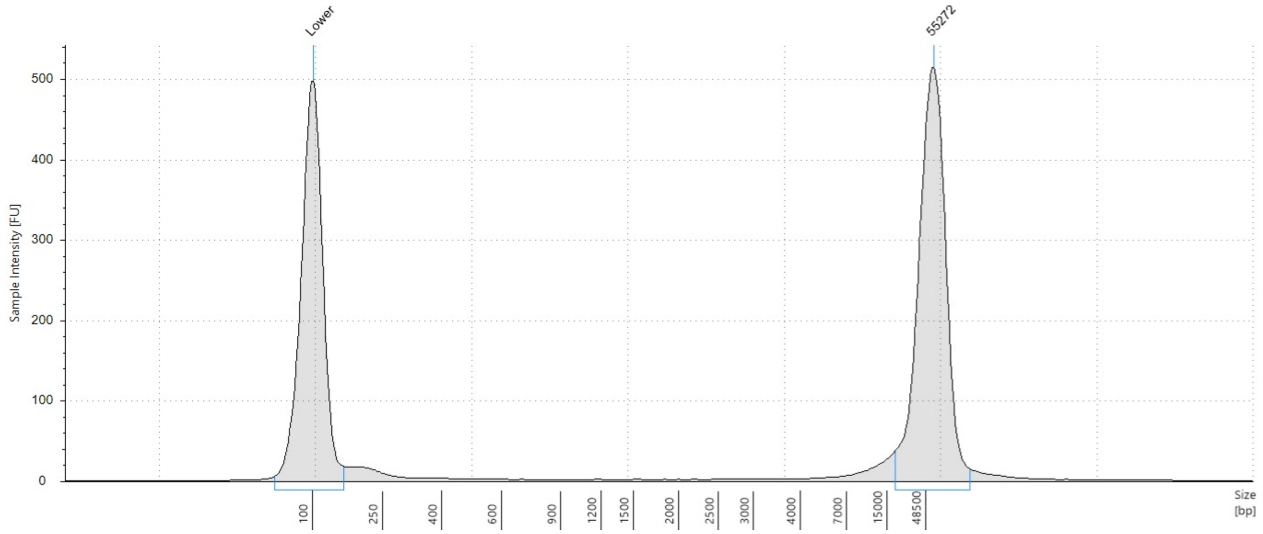

b

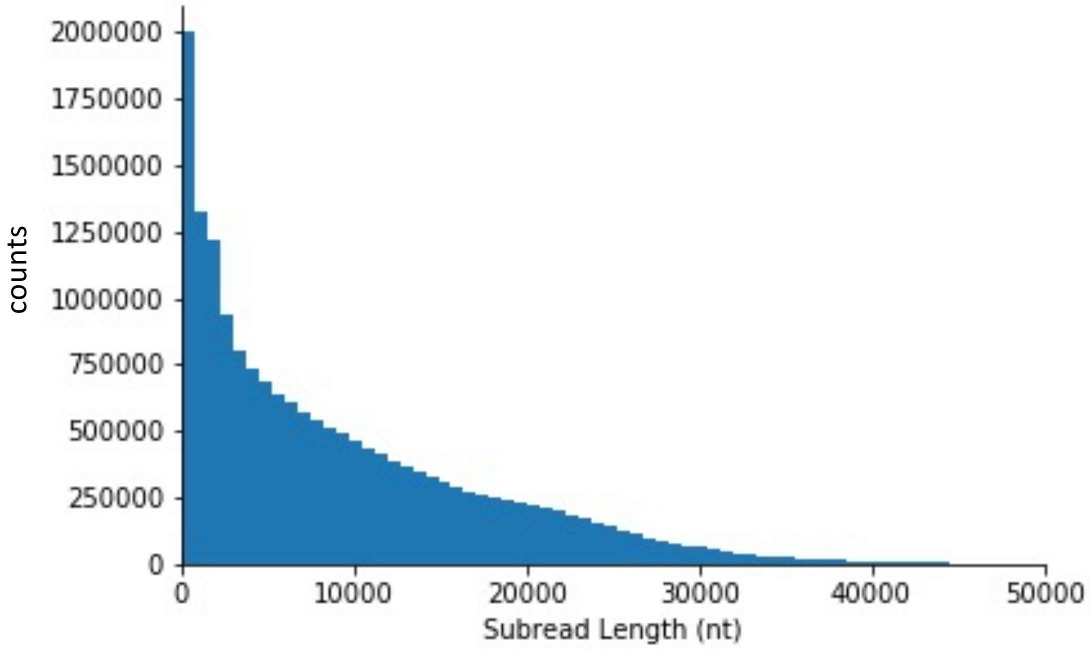

Supplemental Figure 1

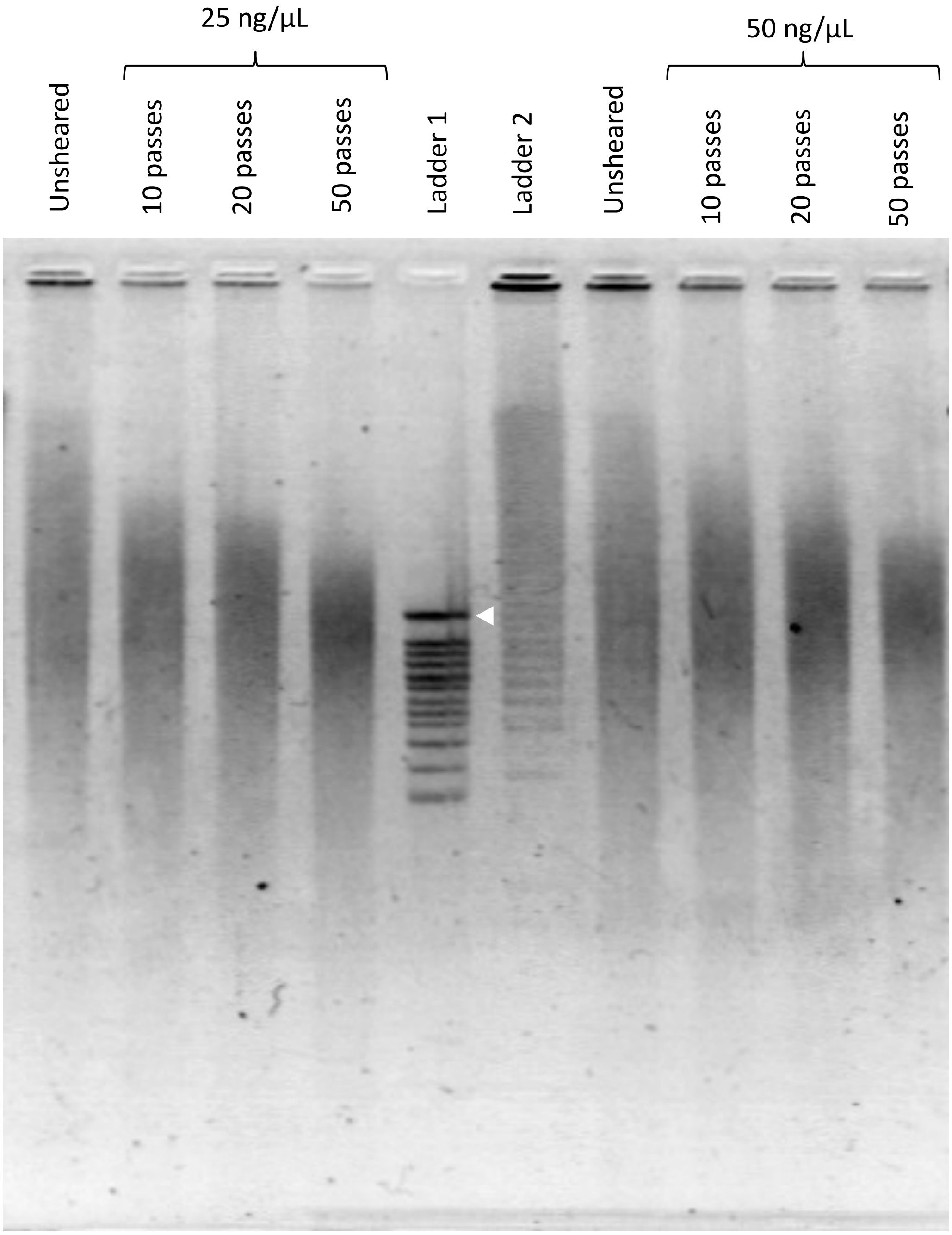

Supplemental Figure 2

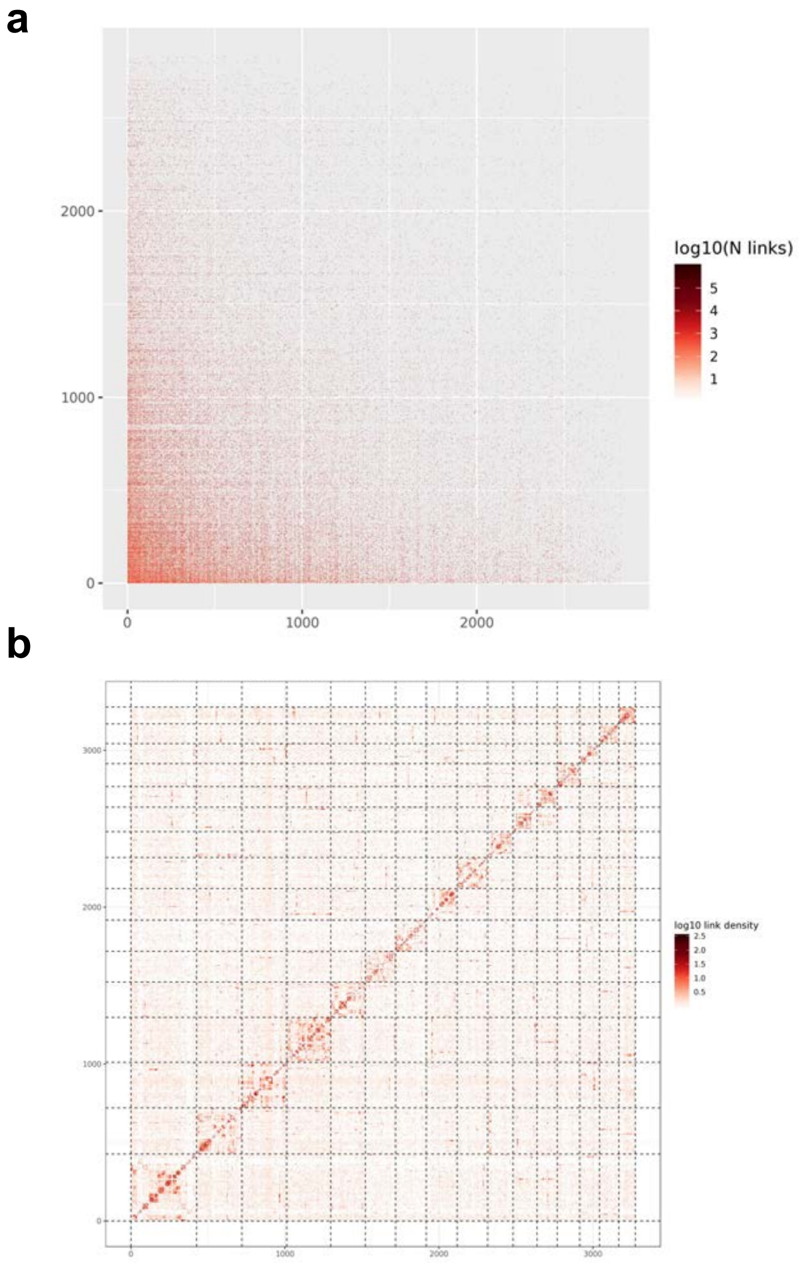

Supplemental Figure 3

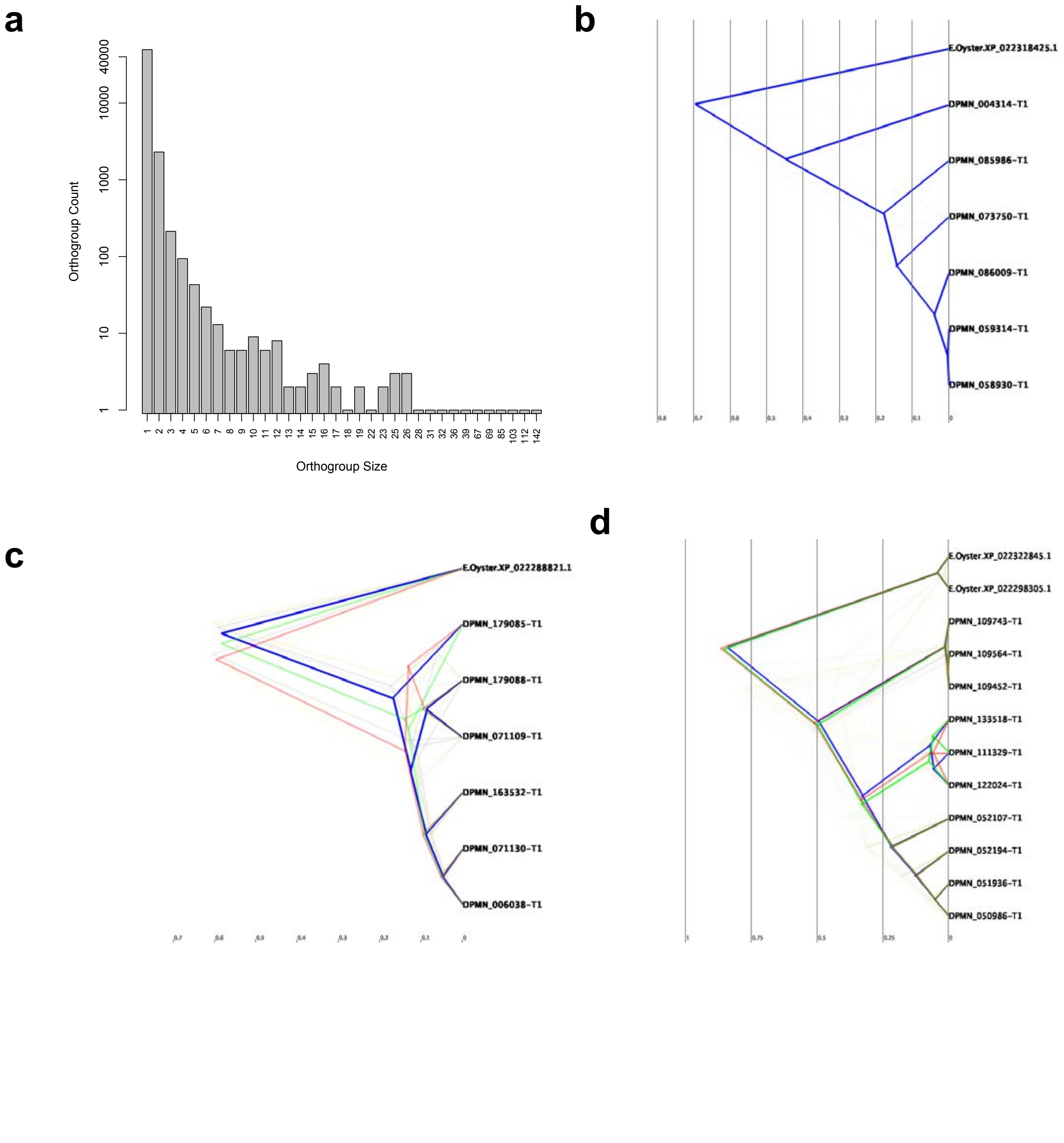

Supplemental Figure 4

a

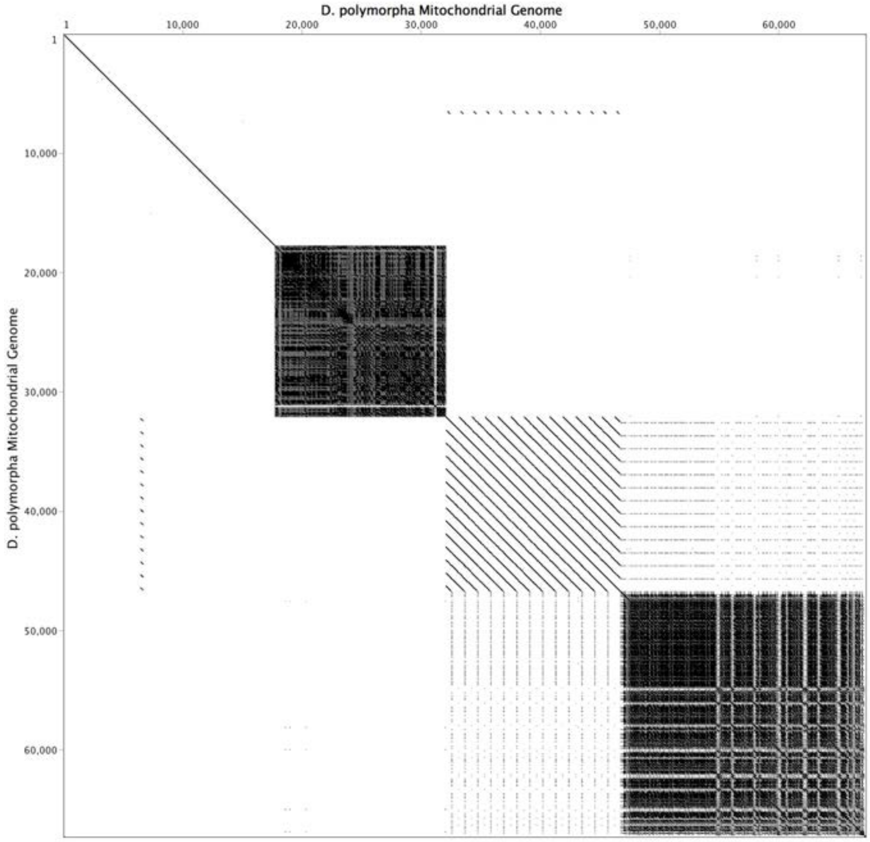

b

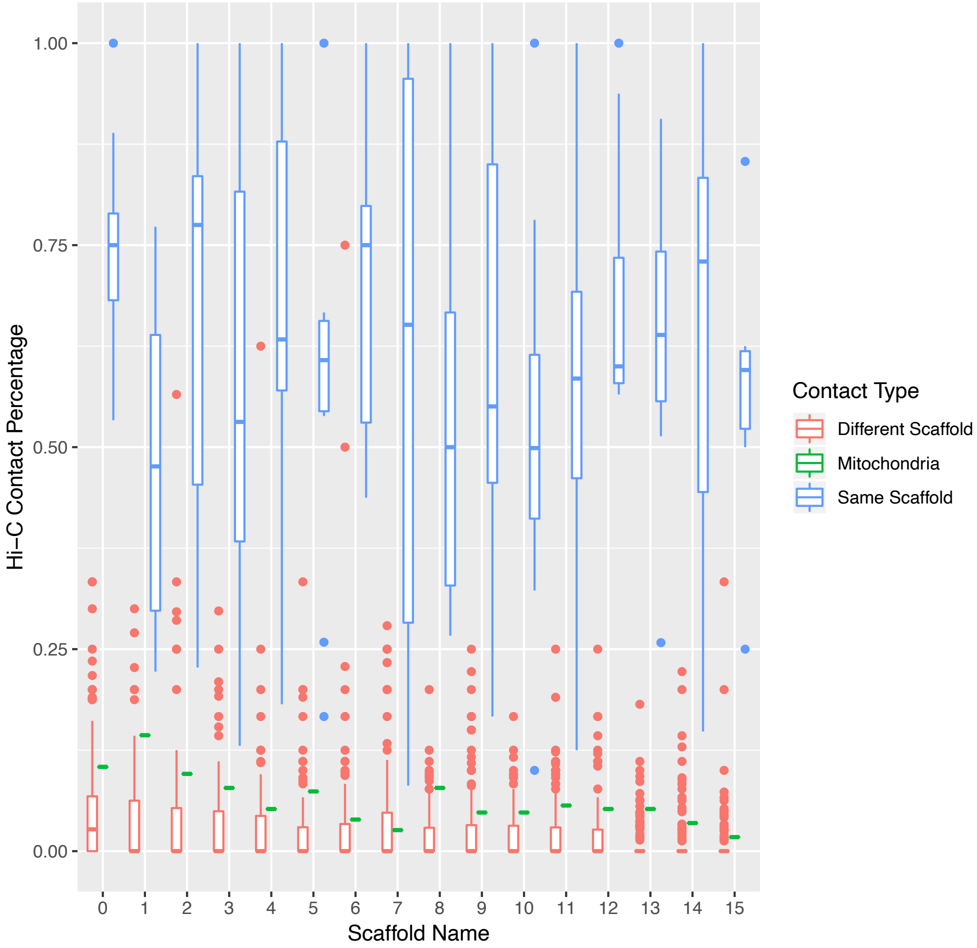

Supplemental Figure 5

a

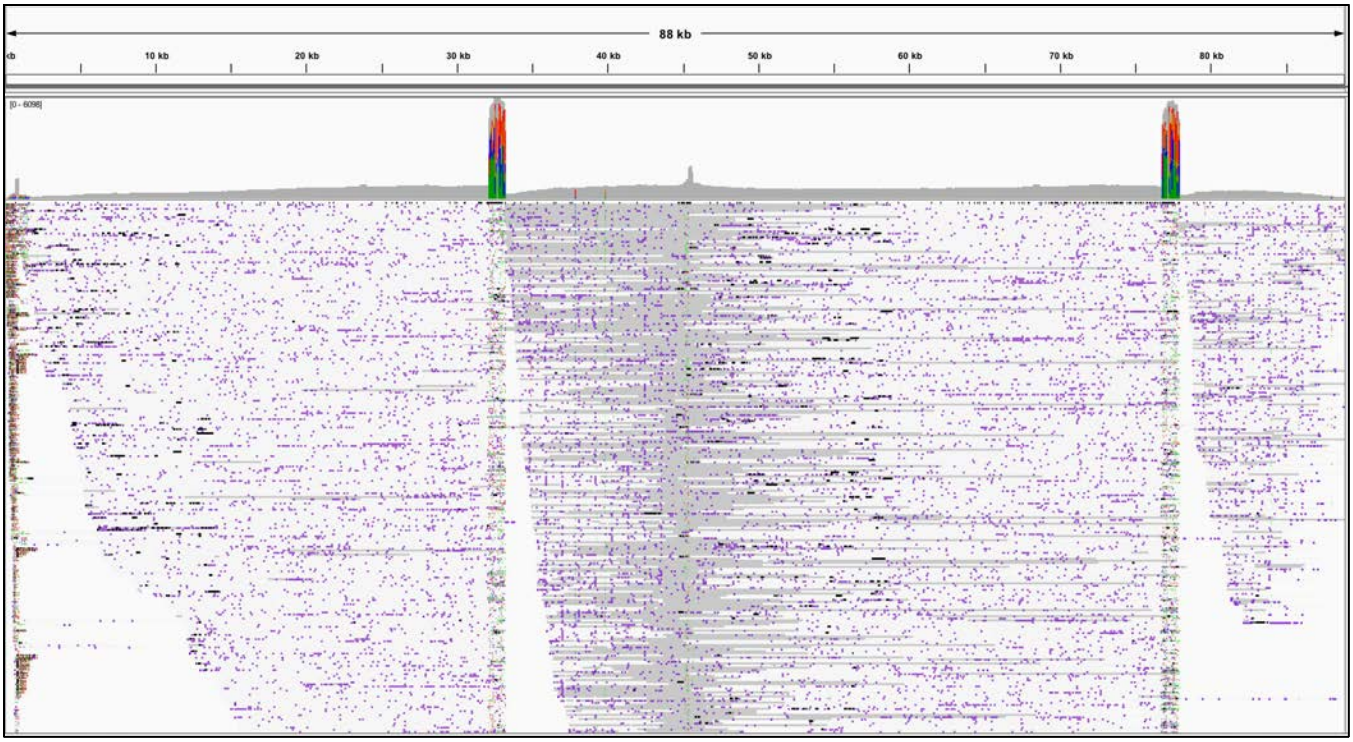

b

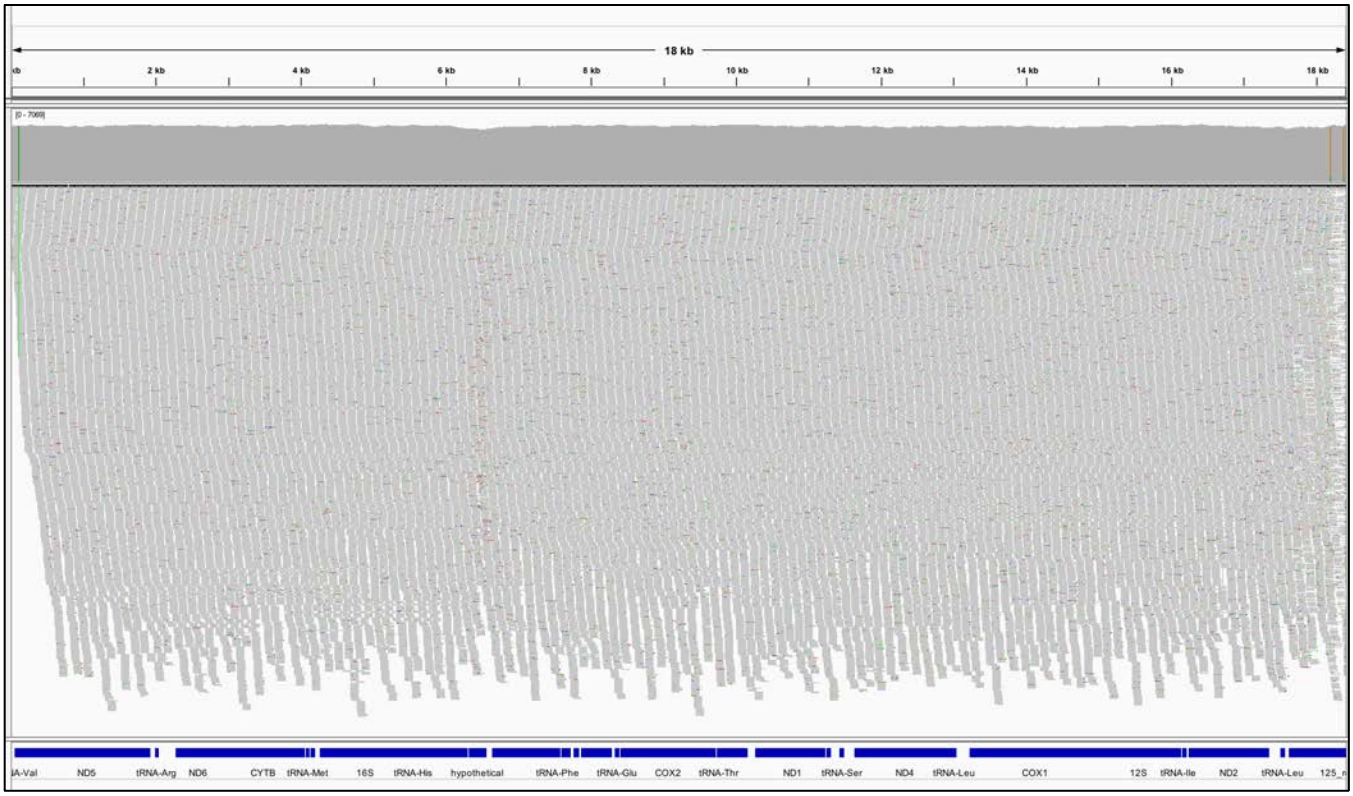

Supplemental Figure 6

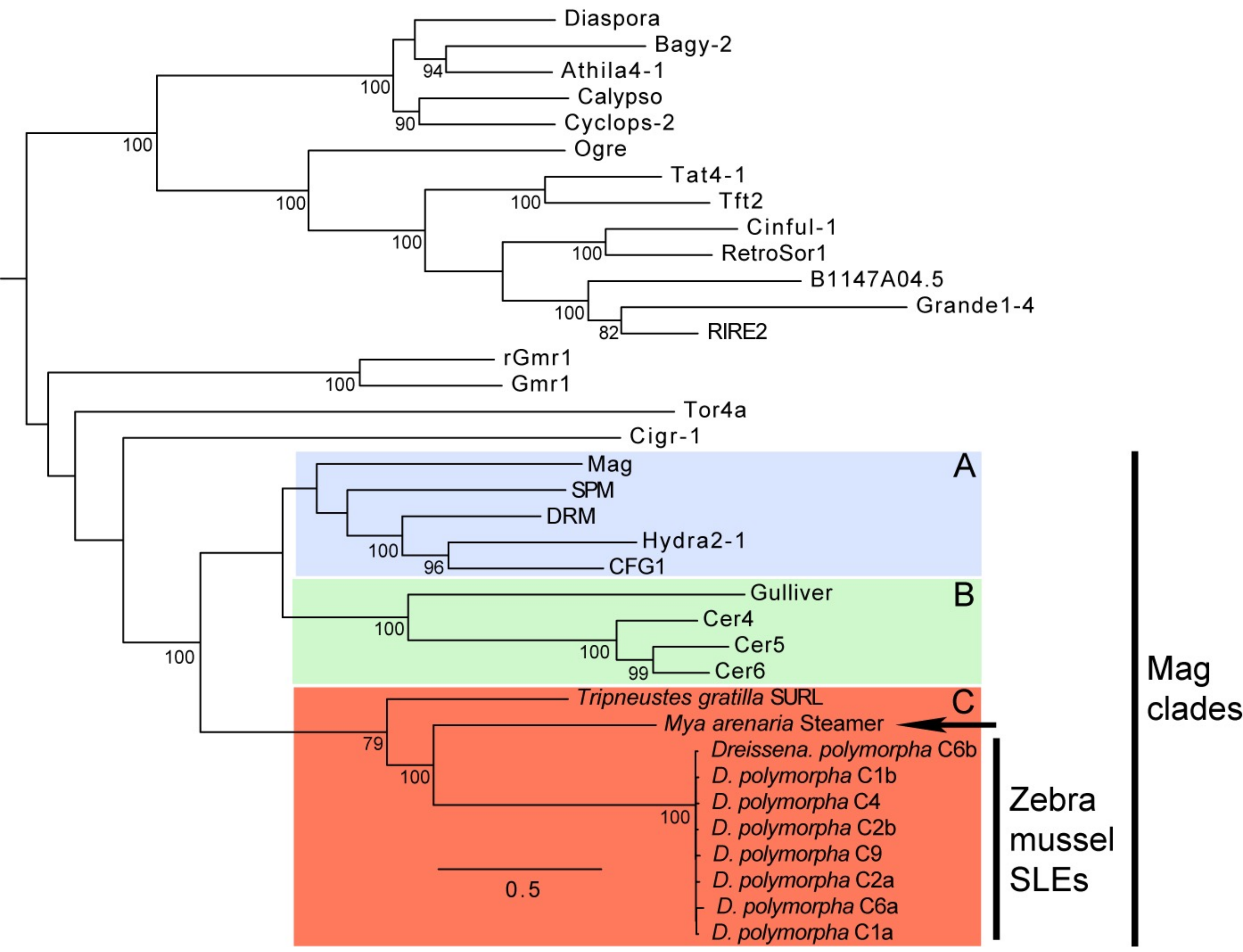

Supplemental Figure 7

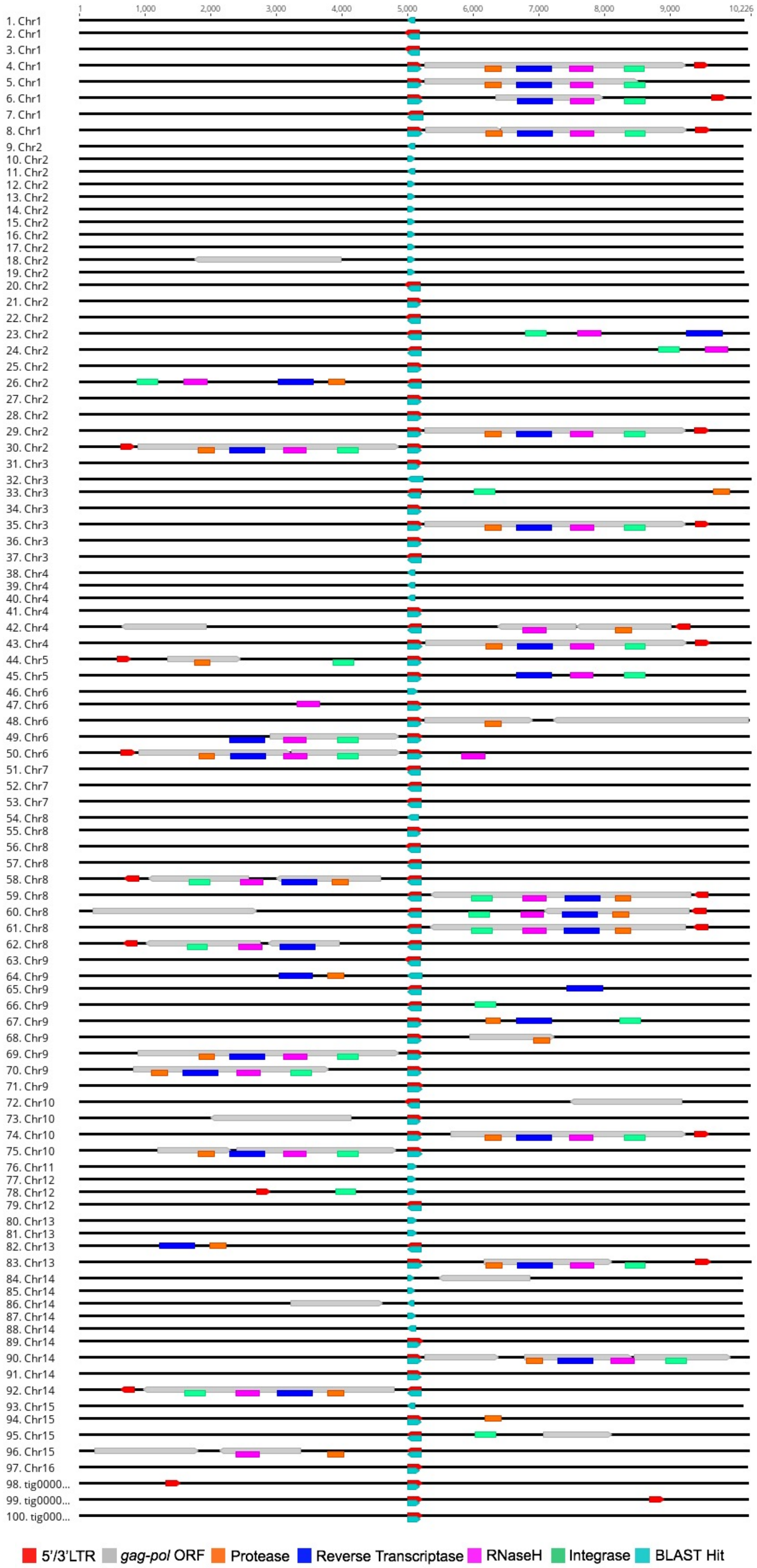

Supplemental Figure 8

a

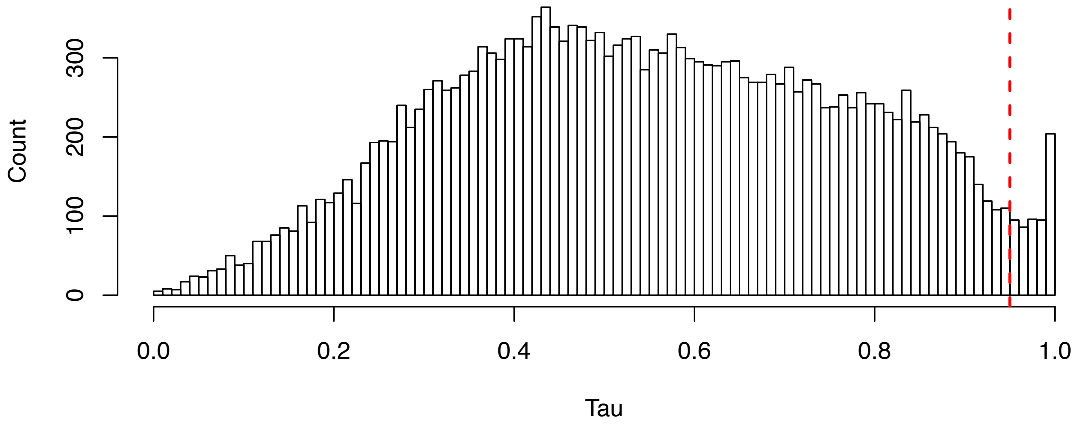

b

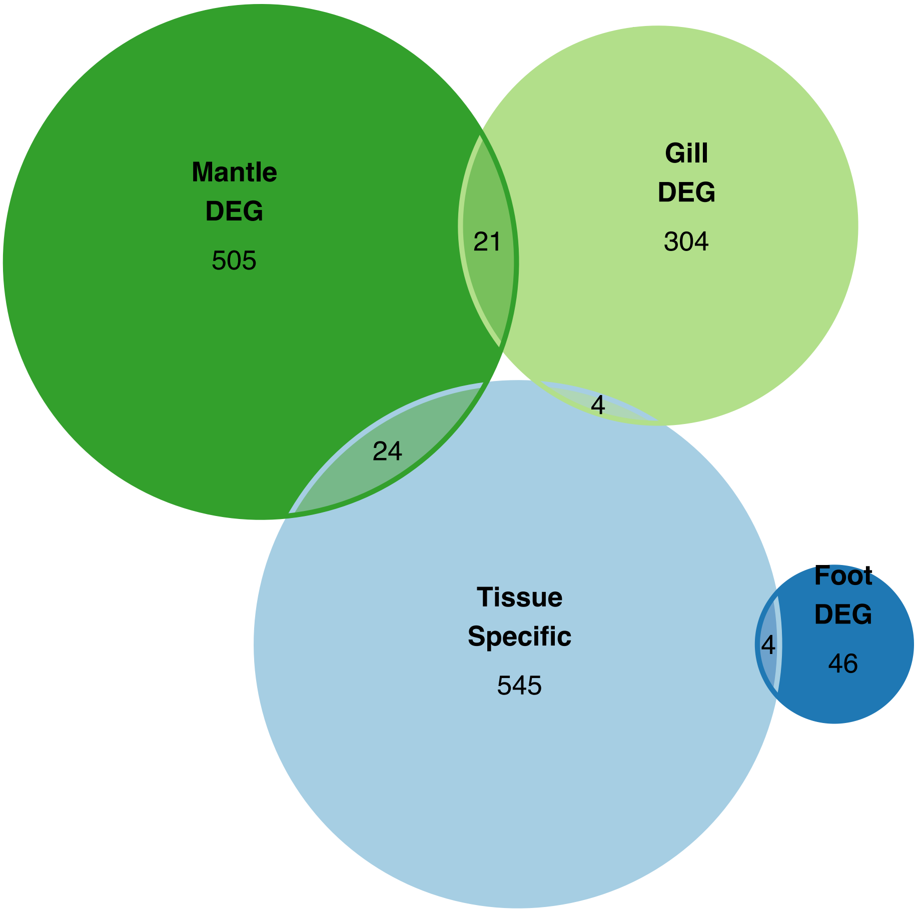

Supplemental Figure 9

```
DPMN_136646      MANQKVALLLIGAVLFAAIGGLDAQGGGFGGGLVGGLVLGGLLEGGLGGGLGGY------   54
DPMN_014835      -MKTSIALLL-AVFAVSAVSGIAKK--GYGGGYGGGYGGGGGGYGGYGGGGGGYGGYGGG   56
DPMN_048164      ----MKSLAL-IALLVGAVVATP-H--GYGHGYGYGSGL-------YGGGGGGYGGYGG-   44
DPMN_173653      -MNTFVALAL-SCVLLSTAFAVR-K-----KGFWWGG-G-------HGGGYGYGAGYGGG   44
DPMN_173639      -MNACVALAV-SCVLLCTVFAVP-K-----KGHGYVG-G-------YGGGYGGYGGYGGS   44
DPMN_126617      -MNACVALAV-SCVLLCTVFAVP-K-----KGHGYGGGG-------YGGGYGGYGGYGGS   45
                       :* :   . . :  .   :      *               *** *

DPMN_136646      ------------GYGGYGFRPPFFGGRFGYPA------------------------YGYG   78
DPMN_014835      YGGGYGGGYGGGDGGGYGGGSFYGGGGLGGGDYGGKKGCHGKHCAFGGGSGGFLGGYGGG   116
DPMN_048164      YGG--------YG--GYG-------------------------------G---LDG----   56
DPMN_173653      YGD--------DG--GYG-------------------------------AVGFAGG----   59
DPMN_173639      YGG--------GD--------------------------------------GYVGG----   54
DPMN_126617      YGG--------GD--------------------------------------GYVGG----   55
                             .

DPMN_136646      FGYPFGGRFF--------------------------------------------------   88
DPMN_014835      YGGGYGGGGYGGG-GYGGGGYGGGGYGGGYGGGEYGGGGLGGYGGGGYGGGYGGGYGGGY   175
DPMN_048164      Y-GGYGGGGYGGYGGFGGYGGYGG-Y----------------------------------   80
DPMN_173653      YGGGYGGG-------Y--GGGYGGGYDDGY------------------------------   80
DPMN_173639      YGGGYGGGGYGGGGGYGGGGYGGGGYGVGY------------------------------   84
DPMN_126617      YGGGYGGGGYGG------------------------------------------------   67
                 :   :**

DPMN_136646      ------------------------------------------------------------   88
DPMN_014835      GGGGYGGKKKCTGKHCGGYGGGGYGGGGYGGGGYGGGGYGGGGYGGGGYGGGYGGGLGGY   235
DPMN_048164      --------------------------------------------GGL---GGY-------   86
DPMN_173653      -------------------------------------GYGGGSFSGVGFGGGYGGGYGGY   103
DPMN_173639      -------------------------------------GYGGGFDGGVGFGGGDGGYGGGF   107
DPMN_126617      -----------------------------------------------------GGYGGGF   74


DPMN_136646      ------------------------------------------------------------   88
DPMN_014835      GGGSYGLGGGGGLGGGGGGFGGVGGFGGSFGHTRQCKCAKRCNKWQKFVGRCPWCQKKCK   295
DPMN_048164      -G-------------GYGGFGHGHGHGWWPHHK---KWRP--------------------   109
DPMN_173653      GG-------------GYGGFGWGN---W--GIRAQCKCAPRCGKFQKYVGRCPWCKKGCK   145
DPMN_173639      GG-------------GYGGYGWGG---KKPTMRAQCKCVKKCGKFQKYVGRCPWCKKGCK   151
DPMN_126617      GG-------------GYGGYGWGG---KKPTMRAQCKCVPKCGKFQKYVGRCPWCKKGCK   118


DPMN_136646      ----------     88
DPMN_014835      LVFCCNRGKY     305
DPMN_048164      ------RKWY     113
DPMN_173653      LVFCCRRKW-     154
DPMN_173639      LVFCCRRKWP     161
DPMN_126617      LVFCCRRKWP     128
```

Supplemental Figure 10

a. DPMN 014835

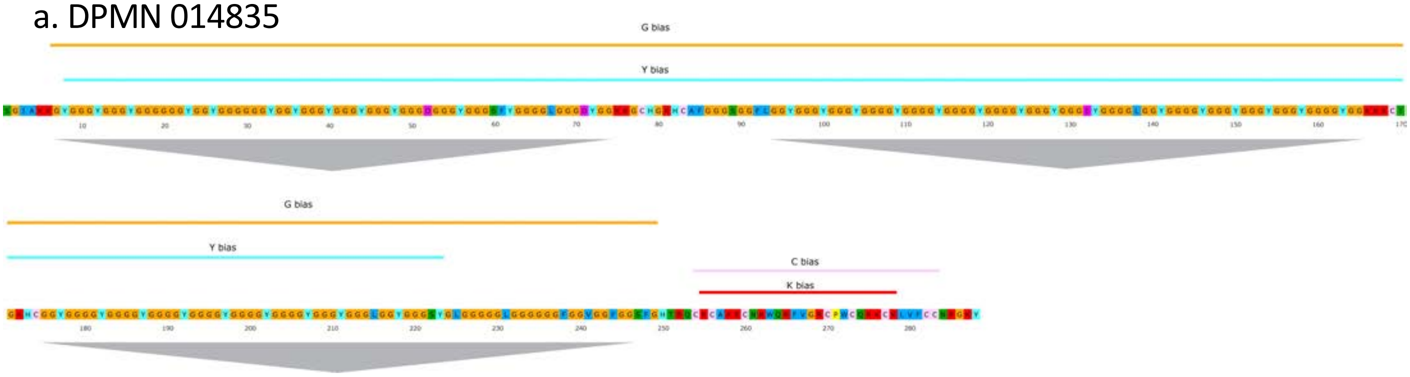

b. DPMN 173639

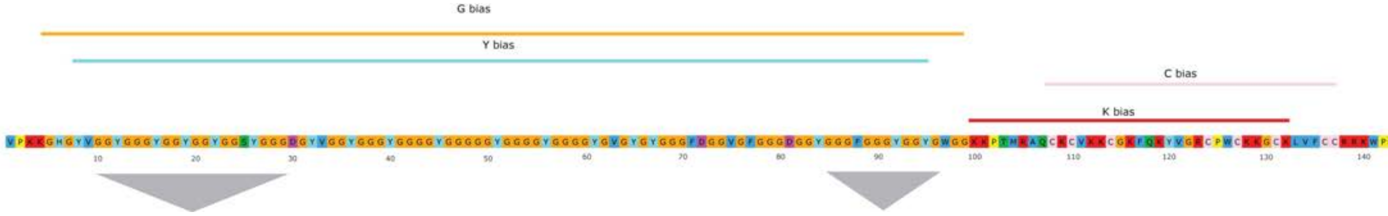

c. DPMN 173653

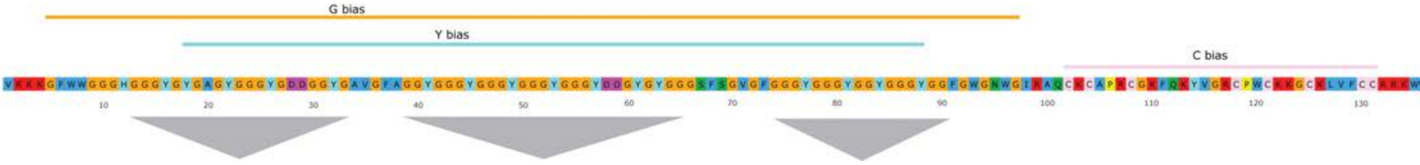

Supplemental Figure 11

d. DPMN 126617

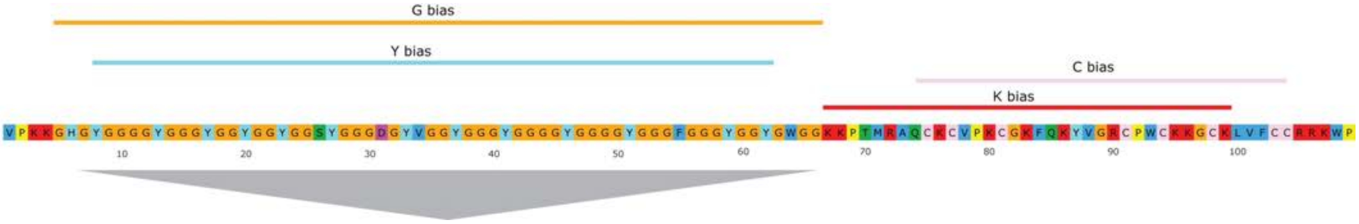

e. DPMN 048164

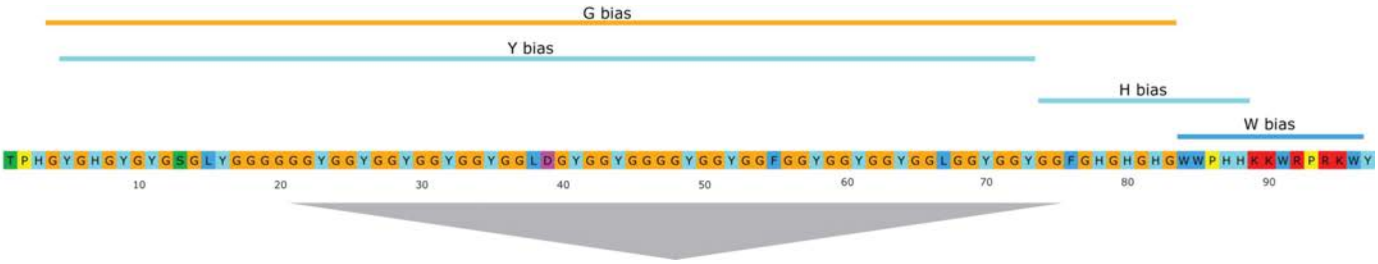

f. DPMN 136646

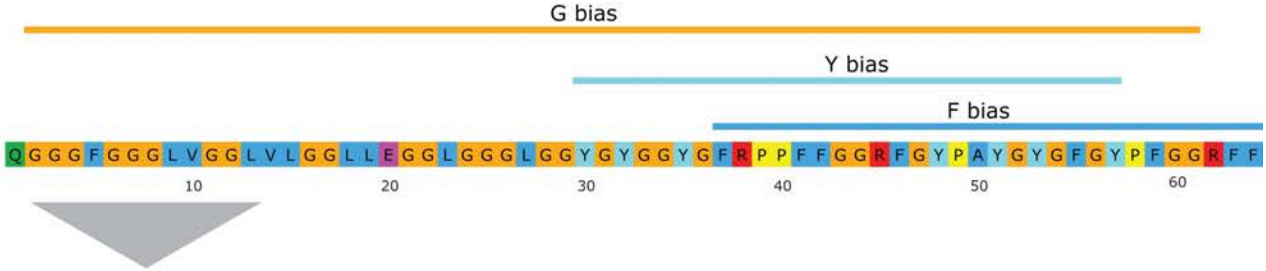

Supplemental Figure 11

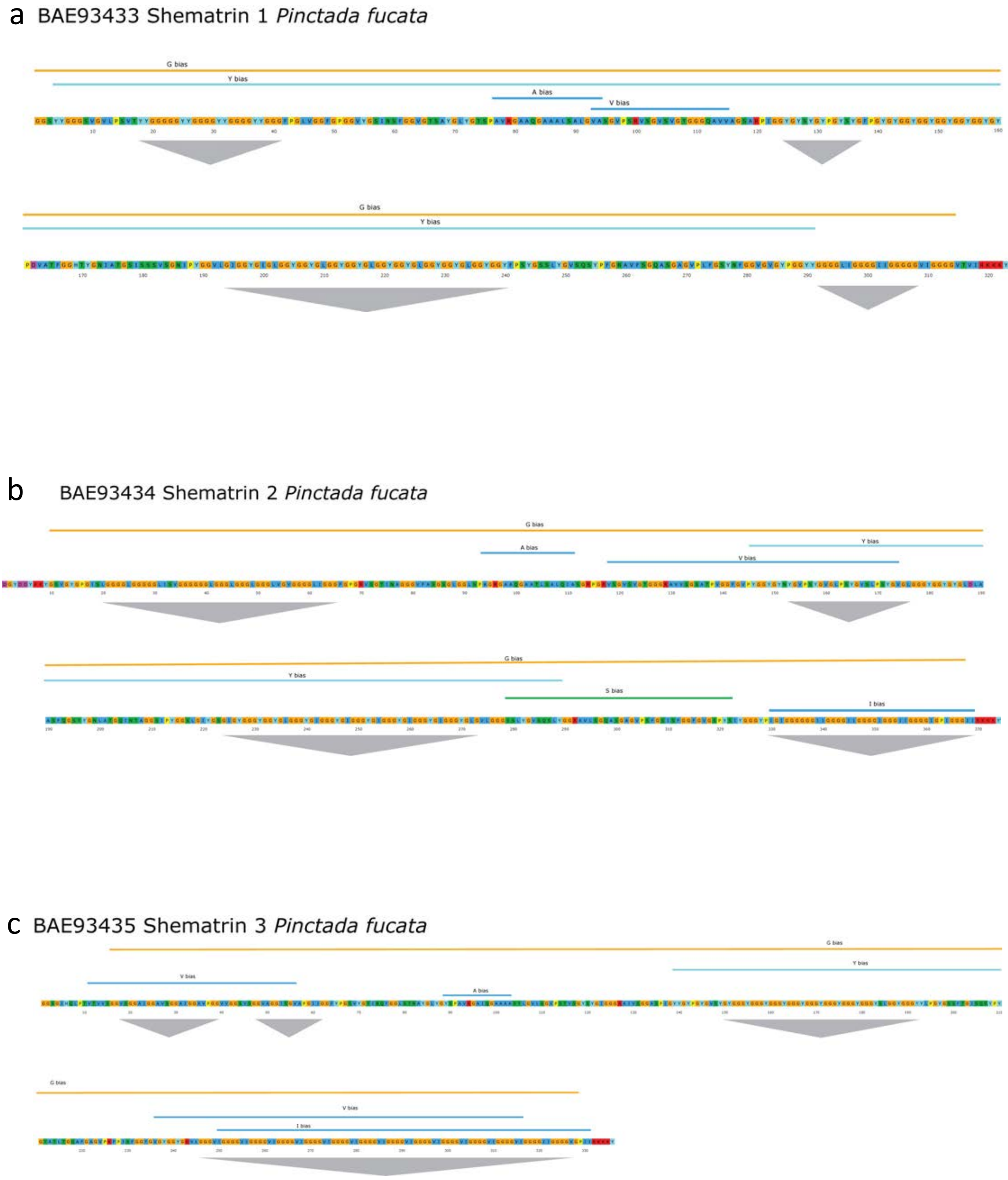

Supplemental Figure 12

d BAE93436 Shematrin 4 *Pinctada fucata*

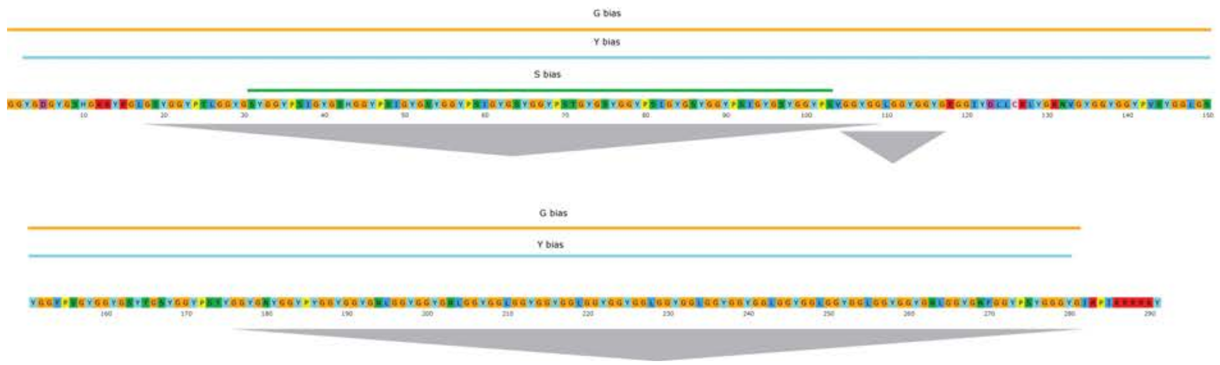

e BAE93437 Shematrin 5 *Pinctada fucata*

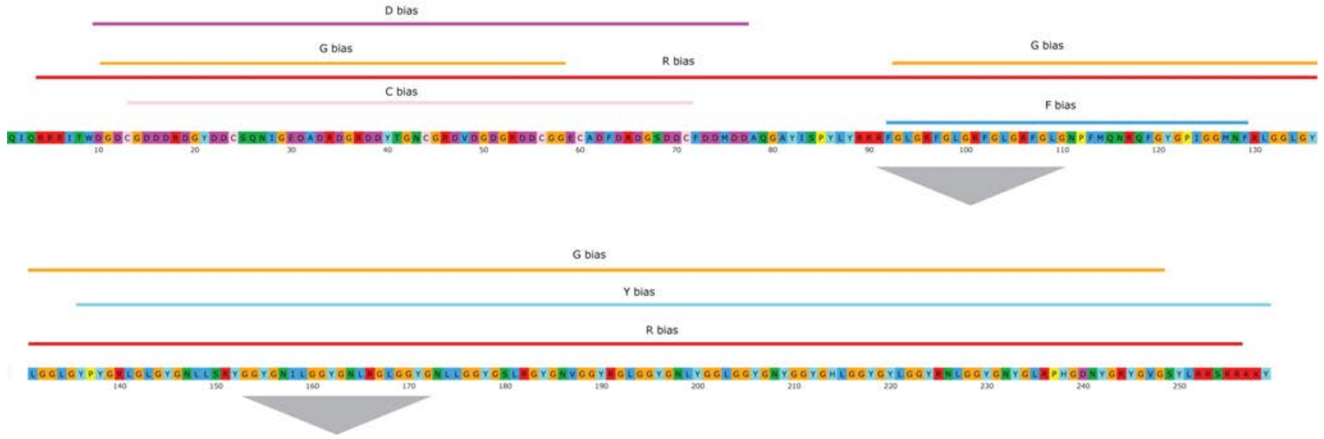

f BAE93438 Shematrin 6 *Pinctada fucata*

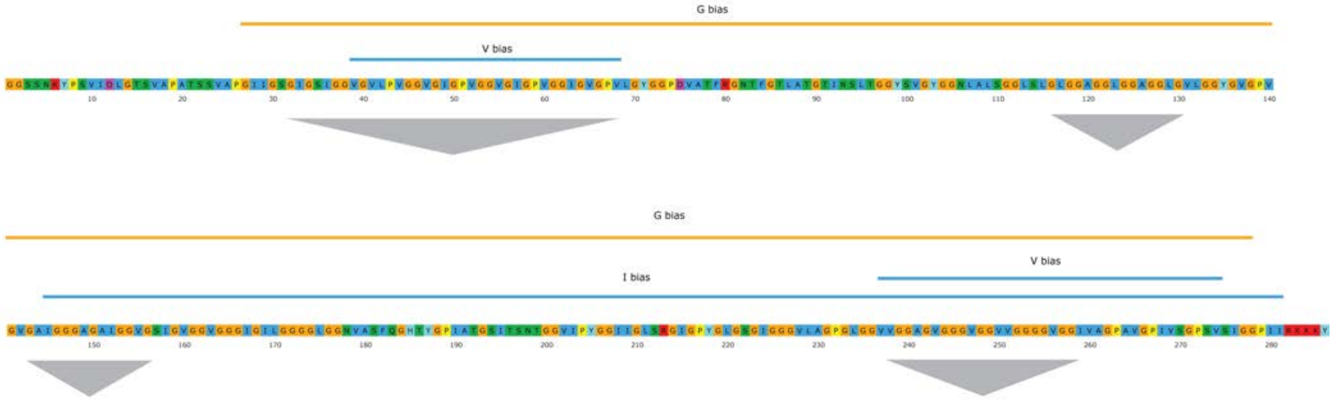

Supplemental Figure 12

g BAE93439 Shematrin 7 *Pinctada fucata*

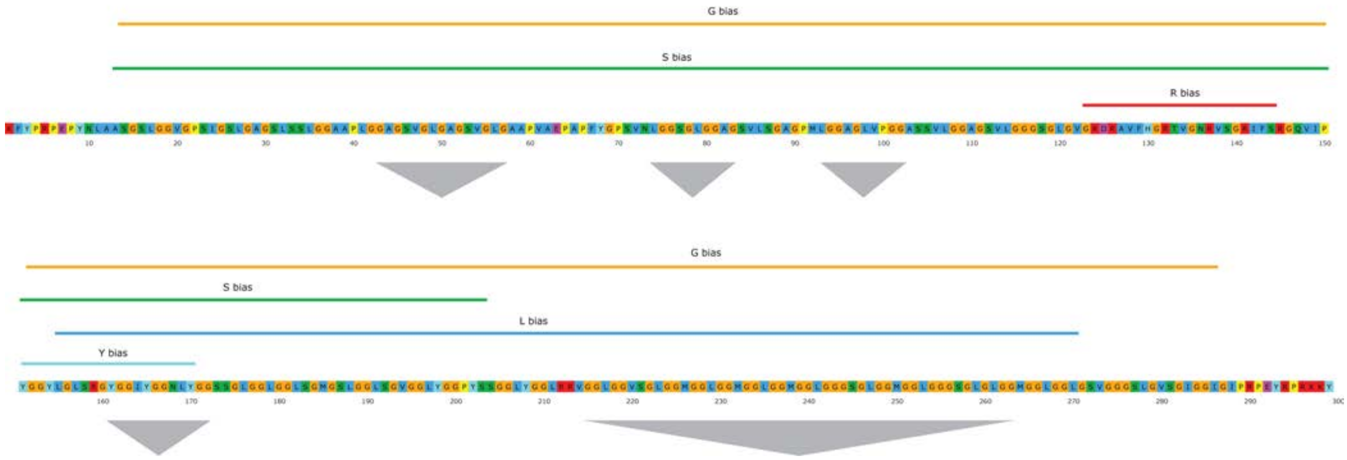

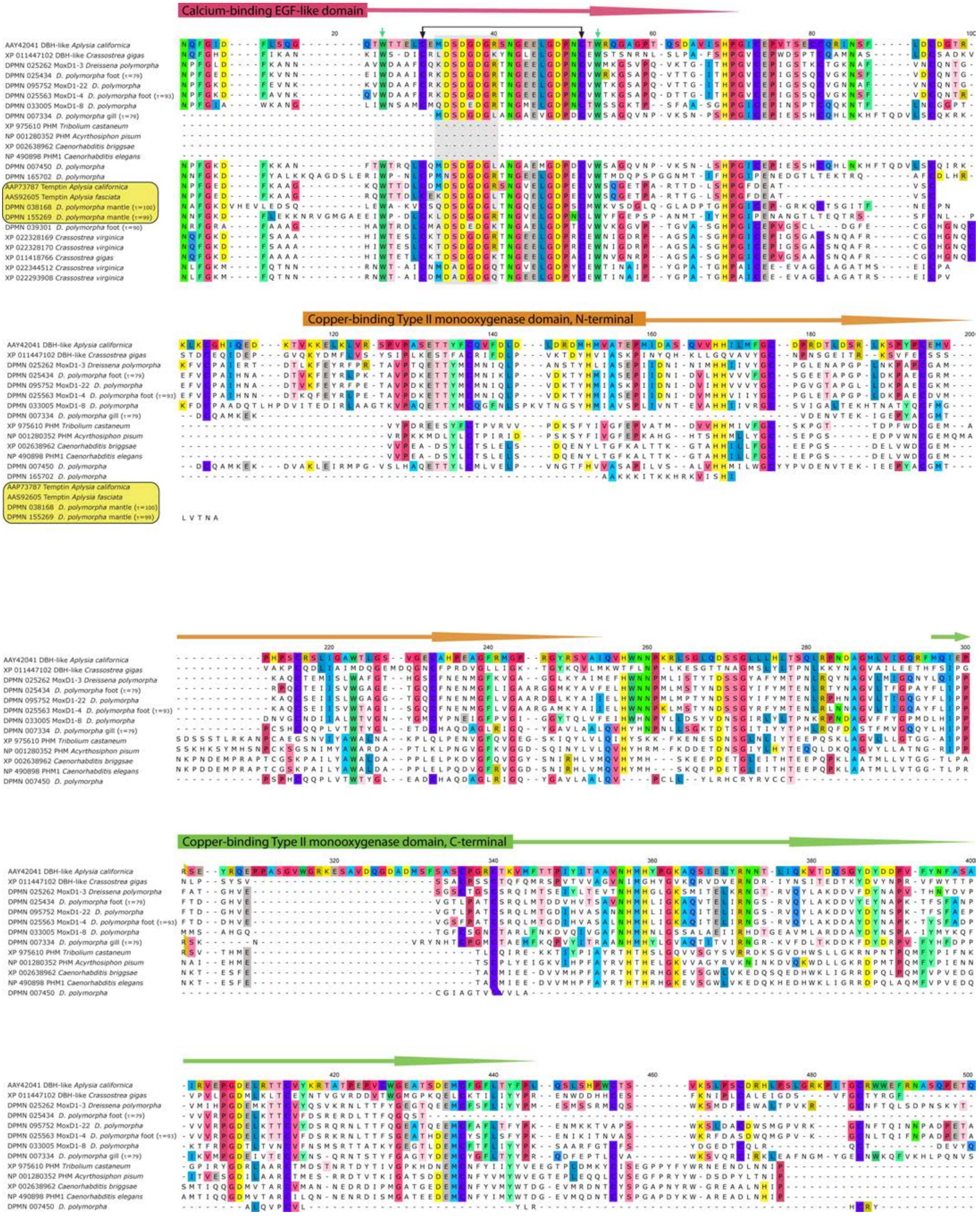

Supplemental Figure 13

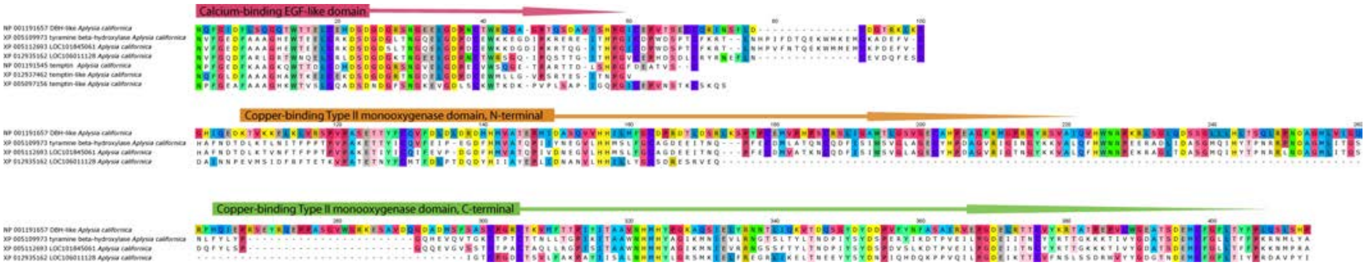

Supplemental Figure 14

a

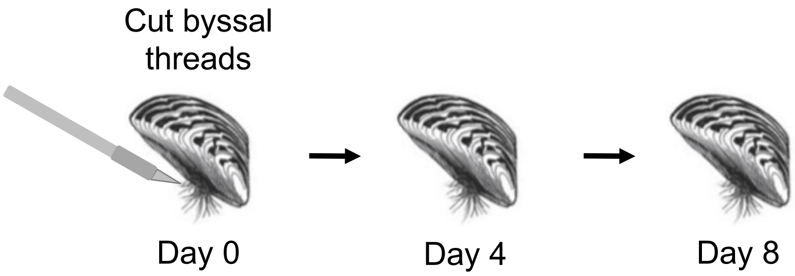

b

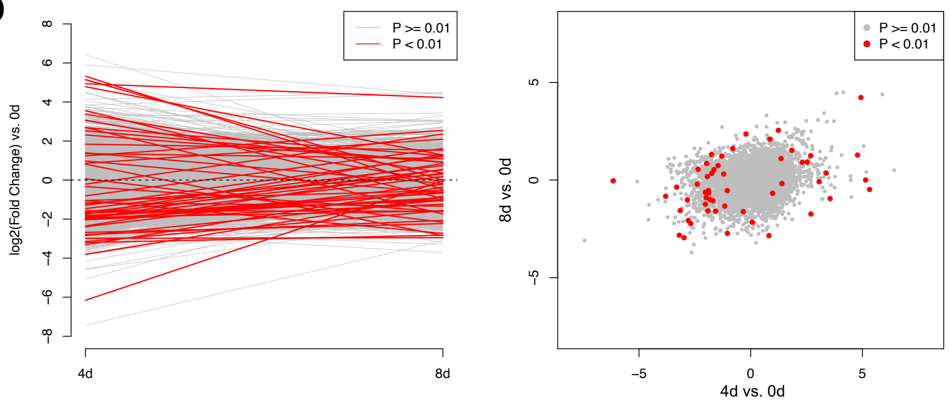

c

Up-regulated genes

Gene ID	BLAST Annotation	Log2FC (4d)
DPMN_066420	Polyadenylate-binding protein 1	5.32
DPMN_017751	Tax1BP1	5.15
DPMN_005570	Tetraspanin	4.93
DPMN_049419	Vitellogenin	4.78
DPMN_134996	Glutamyl aminopeptidase-like	3.56
DPMN_013285	Monocarboxylate transporter 12	3.38
DPMN_034632	CD109	3.07
DPMN_125763	CD109	2.69
DPMN_011081	Tenascin-X	2.68
DPMN_080226	Metabotropic glutamate receptor	2.53
DPMN_194568	60S ribosomal protein L19	2.31
DPMN_066988	Serine/threonine-protein kinase	1.84
DPMN_013817	Kyphoscoliosis peptidase	1.40
DPMN_027392	Anoctamin-4-like	1.36
DPMN_121885	Arylsulfatase	1.24
DPMN_164933	Cytochrome P450 3A28-like	0.98
DPMN_127641	Cytosolic Fe-S cluster assembly	0.86
DPMN_053135	Ceroid-lipofuscinosis; neuronal 5	0.82
DPMN_122749	Lysocardiolipin acyltransferase 1	0.07

Down-regulated genes

Gene ID	BLAST Annotation.	Log2FC (4d)
DPMN_181702	Nucleoside diphosphate kinase	-6.16
DPMN_092058	No similarity found	-3.80
DPMN_114486	Metalloreductase STEAP2-like	-3.31
DPMN_103363	Alpha-tubulin	-3.20
DPMN_028019	von Willebrand factor D and EGF	-3.15
DPMN_084917	AMBP	-2.98
DPMN_186960	Toll-like receptor 4	-2.84
DPMN_030517	von Willebrand factor D and EGF	-2.79
DPMN_118567	Cytokine receptor	-2.68
DPMN_187139	CXorf38 homolog	-2.39
DPMN_114830	Serine/threonine phosphatase	-2.35
DPMN_106288	No similarity found	-2.03
DPMN_097756	No similarity found	-2.03
DPMN_085219	Complement factor B-like protein	-2.00
DPMN_062091	Solute carrier family 23 member	-1.97
DPMN_028623	Matrix metallopeptidase 10	-1.94
DPMN_084246	TNF ligand-like	-1.91
DPMN_004625	DUF4921-domain-containing	-1.90
DPMN_010384	Major facilitator super domain	-1.88
DPMN_102295	Bcl2-like 1	-1.82

Supplemental Figure 15

	Species	Family	Common name	Economic significance	Assembly level	Number of scaffolds	Number of contigs	Contig N50 (bp)	Genome length (Mb)	Reference
1	*Archivesica marissinica*	Vesicomyidae	Deep sea chemosymbiotic clam	None	Chromosome	4,005	29,278	79,144	1,544	Ip et al. 2021
2	*Argopecten irradians concentricus*	Pectinidae	Bay scallop	Wild harvest and hatchery culture	Scaffold	82,208	119,459	63,725	875	Liu et al. 2020
3	*Chlamys farreri*	Pectinidae	Zhikong (Chinese) scallop	Wild harvest and culture	Scaffold	96,024	148,999	21,500	780	Li et al. 2017
4	*Crassostrea gigas*	Ostreidae	Pacific oyster	Hatchery culture—leads aquatic animals in global harvest	Scaffold	7,659	30,460	31,239	558	Zhang et al. 2012
5	*Crassostrea virginica*	Ostreidae	Eastern oyster	Wild harvest and hatchery culture	Chromosome	11	669	1,971,208	685	Gómez-Chiarri et al. 2015
6	*Cyclina sinensis*	Veneridae	Venus clam	Hatchery culture	Scaffold	187	701	2,587,078	903	Wei et al. 2020
7	*Dreissena rostriformis*	Dreissenidae	Quagga mussel	Invasive	Scaffold	18,514	49,413	45,905	1,241	Calcino et al. 2019
8	*Gigantidas platifrons*	Mytilidae	Hydrothermal vent mussel	None	Scaffold	65,662	272,497	12,602	1,658	Sun et al. 2017
9	*Limnoperna fortunei*	Mytilidae	Golden mussel	Invasive	Scaffold	20,580	61,104	32,203	1,673	Uliano-Silva et al. 2018
10	*Magallana hongkongensis*	Ostreidae	Hong Kong oyster	Hatchery culture	Scaffold	11,926	98,303	17,202	7,579	Li et al. 2020
11	*Margaritifera margaritifera*	Margaritiferidae	Freshwater pearlshell mussel	Historically, pearl harvest	Scaffold	105,182	289,398	16,891	2,472	Gomes-dos-Santos et al. 2021
12	*Megalonaias nervosa*	Unionidae	Washboard mussel	None	Scaffold	96,310	97,291	50,186	2,365	Rogers et al. 2020
13	*Mercenaria mercenaria*	Veneridae	Hard clam	Wild harvest and hatchery culture	Chromosome	1,537	4,218	1,773,430	1,788	Song et al. 2021
14	*Mizuhopecten yessoensis*	Pectinidae	Yesso scalllop	Culture from wild seed	Scaffold	82,659	120,022	65,014	988	Wang et al. 2017
15	*Modiolus philippinarum*	Mytilidae	Phillipine horse mussel	None	Scaffold	74,573	301,873	18,389	2,630	Sun et al. 2017
16	*Mytilus corsucus*	Mytilidae	Hard shelled mussel	Wild harvest and hatchery culture	Chromosome	4,434	6,449	1,481,111	1,566	Yang et al. 2021
17	*Mytilus edulis*	Mytilidae	Blue mussel	Wild harvest and hatchery culture	Scaffold	3,339	5,966	511,485	1,827	Unpublished (2021)
18	*Mytilus galloprovincialis*	Mytilidae	Mediterranean mussel	Wild harvest and hatchery culture	Scaffold	10,577	22,883	77,157	1,282	Gerdol et al. 2020
19	*Ostrea lurida*	Ostreidae	Olympia oyster	Historically, wild harvest	Scaffold	159,429	466,560	7,815	1,140	Unpublished (2020)
20	*Panopea generosa*	Hiatellidae	Pacific geoduck clam	Wild harvest and hatchery culture	Scaffold	18	113,179	14,495	942	Unpublished (2020)
21	*Pecten maximus*	Pectinidae	Great scallop	Wild harvest and hatchery culture	Chromosome	3,983	5,809	1,258,799	918	Kenny et al. 2020
22	*Perna viridis*	Mytilidae	Green mussel	Wild harvest and hatchery culture	Scaffold	15,933	39,299	70,096	731	Inoue et al. 2021
23	*Pinctada imbricata*	Pteriidae	Akoya pearl oyster	Cultured pearls	Chromosome	5,039	85,944	21,518	991	Du et al. 2017
24	*Pinna nobilis*	Pinnidae	Noble pen shell	Source of sea silk (prior to 20th century)	Scaffold	97,633	147,400	5,822	5,864	Unpublished (2020)

Supplemental Table 1

25	*Potamilus streckersoni*	Unionidae	Brazos heelsplitter	None	Scaffold	2,366	2,460	2,032,685	1,777	Smith 2021
26	*Ruditapes philippinarum*	Veneridae	Manila clam	Major species in culture worldwide	Chromosome	30,670	85,994	29,238	1,123	Yan et al. 2019
27	*Saccostrea glomerata*	Ostreidae	Sydney rock oyster	Wild harvest and hatchery culture	Scaffold	10,101	39,811	39,540	788	Powell et al. 2018
28	*Sinonovacula constricta*	Pharidae	Chinese razor clam	Major species in culture worldwide	Chromosome	362	2,450	976,936	1,220	Ran et al. 2019
29	*Tegillarca granosa*	Arcidae	Blood clam	Cultured for food	Scaffold	269	2,112	605,873	797	Bao et al. 2021
30	*Venustaconcha ellipsiformis*	Unionidae	Ellipse	None	Scaffold	371,427	820,906	2,813	1,590	Renaut et al. 2018

```
==================================================
file name: PGA_Assembly.FINAL.fasta
sequences:           145
total length: 1798019516 bp  (1797746116 bp excl N/X-runs)
GC level:         35.14 %
bases masked:  852870596 bp ( 47.43 %)
==================================================
               number of      length   percentage
               elements*    occupied  of sequence
--------------------------------------------------
SINEs:            50552     11629297 bp    0.65 %
      ALUs            0            0 bp    0.00 %
      MIRs        12195      1557593 bp    0.09 %

LINEs:           115027     77616133 bp    4.32 %
      LINE1        4532      2311960 bp    0.13 %
      LINE2        9224      4723805 bp    0.26 %
      L3/CR1       6363      4054095 bp    0.23 %

LTR elements:     38917     27955936 bp    1.55 %
      ERVL           35         8358 bp    0.00 %
      ERVL-MaLRs      1          120 bp    0.00 %
      ERV_classI    827       507019 bp    0.03 %
      ERV_classII   397        26602 bp    0.00 %

DNA elements:    244050     80624865 bp    4.48 %
     hAT-Charlie    773       239831 bp    0.01 %
     TcMar-Tigger   635       120753 bp    0.01 %

Unclassified:    2068170    617626932 bp   34.35 %

Total interspersed repeats:815453163 bp   45.35 %


Small RNA:         5444       693184 bp    0.04 %

Satellites:        8601      1869040 bp    0.10 %
Simple repeats:  316718     37443122 bp    2.08 %
Low complexity:   29370      1463637 bp    0.08 %
==================================================
```

Supplemental Table 2
