# Supplementary material for: The genome of the zebra mussel, Dreissena polymorpha: a resource for comparative genomics, invasion genetics, and biocontrol
Source: G3 (Bethesda). 2021 Dec 13;12(2):jkab423. doi: 10.1093/g3journal/jkab423 (PMC9210306; doi:10.1093/g3journal/jkab423)
Supplement: jkab423_Supplementary_Files [file jkab423_supplementary_files.zip › Supplemental info/13. Byssalome.docx]

**Chromosomal locations of byssal genes in *D. polymorpha.*** Mb = megabases. Length cds = length (bp) of the coding DNA sequence. Cds were resolved using SPLIGN. Gene IDs are from our automated annotation, NA = not annotated. * The region corresponding to Dpfp8 is at the C-terminus of two longer ORFs. DPMN_074964 is 7194 bp, and DPMN_074797 is 1881 bp.

| **Byssal protein(s)** | **Locus** | **Chr** | | **Strand** | | **Chr L (Mb)** | | **Length cds** | **Number of exons** | | **Gene ID** |
| --- | --- | --- | --- | --- | --- | --- | --- | --- | --- | --- | --- |
| Dpfp7beta | 1 | 1 | | + | | 190.57 | | 1308 | 3 | | DPMN 059800 |
| Dpfp9beta | 1 | 2 | | + | | 173.08 | | 278 | 1 | | DPMN 170509 |
| Dpfp9beta | 2 | 2 | | + | | 173.08 | | 273 | 1 | | NA |
| Dpfp8 | 1 | 3 | | - | | 148.54 | | 195 | 1 | | DPMN 074964* |
| Dpfp8 | 2 | 3 | | - | | 148.54 | | 195 | 1 | | DPMN 074797* |
| Dpfp11alpha | 2 | 4 | | - | | 144.72 | | 2788 | 3 | | NA |
| Dpfp11alpha | 3 | 4 | | - | | 144.72 | | 2787 | 3 | | NA |
| Dpfp11beta | 1 | 4 | | - | | 144.72 | | 1309 | 3 | | NA |
| Dpfp7beta | 1 | 4 | | + | | 144.72 | | 1258 | 3 | | NA |
| Dpfp9beta | 1 | 5 | | + | | 127.52 | | 270 | 1 | | DPMN 129274 |
| Dpfp9alpha | 2 | 5 | | + | | 127.52 | | 258 | 1 | | DPMN 129263 |
| Dpfp9alpha | 3 | 5 | | + | | 127.52 | | 267 | 1 | | DPMN 129218 |
| Dpfp9alpha | 4 | 5 | | + | | 127.52 | | 264 | 1 | | DPMN 129185 |
| Dpfp9alpha | 5 | 5 | | + | | 127.52 | | 264 | 1 | | DPMN 129124 |
| Dpfp9alpha | 6 | 5 | | + | | 127.52 | | 267 | 1 | | DPMN 129102 |
| Dpfp9alpha | 7 | 5 | | + | | 127.52 | | 267 | 1 | | NA |
| Dpfp9alpha | 8 | 5 | | + | | 127.52 | | 267 | 1 | | DPMN 129080 |
| Dpfp9alpha | 9 | 5 | | + | | 127.52 | | 267 | 1 | | DPMN 129068 |
| Dpfp9beta | 10 | 5 | | + | | 127.52 | | 276 | 1 | | DPMN 129022 |
| Dpfp9beta | 11 | 5 | | + | | 127.52 | | 276 | 1 | | NA |
| Dpfp2 | 1 | | 6 | | + | | 109.03 | 662 | | 3 | DPMN_127665 |
| Dpfp10 | 1 | | 9 | | - | | 98.41 | 1337 | | 3 | DPMN_120898 |
| Dpfp10 | 2 | | 9 | | - | | 98.41 | 1713 | | 3 | NA |
| Dpfp9beta | 1 | | 9 | | + | | 98.41 | 267 | | 1 | DPMN_059300 |
| Dpfp7alpha | 1 | | 11 | | + | | 92.27 | 1295 | | 3 | NA |
| Dpfp7gamma | 1 | | 11 | | - | | 92.27 | 1190 | | 3 | NA |
| Dpfp7alpha | 1 | | 12 | | + | | 85.01 | 1290 | | 3 | NA |
| Dpfp7alpha | 2 | | 12 | | + | | 85.01 | 1289 | | 3 | NA |
| Dpfp7gamma | 1 | | 12 | | - | | 85.01 | 1231 | | 3 | DPMN_136767 |
| Dpfp7gamma | 2 | | 12 | | - | | 85.01 | 1241 | | 3 | NA |
| Dpfp1/Dpfp6 | 1 | | 13 | | + | | 81.15 | 14235 | | 16/9 | NA |
| Dpfp10 | 1 | | 13 | | - | | 81.15 | 1422 | | 3 | DPMN 159905 |
| Dpfp10 | 2 | | 13 | | + | | 81.15 | 851 | | 3 | DPMN 159531 |
| Dpfp10 | 3 | | 13 | | + | | 81.15 | 1421 | | 3 | DPMN 159708 |
| Dpfp5.1 | 1 | | 13 | | + | | 81.15 | 1623 | | 3 | NA |
| Dpfp5.2 | 2 | | 13 | | - | | 81.15 | 1704 | | 3 | NA |

**Table MAM2b. Summary and highlights from the byssalome.**

| **Byssal protein(s)** | **Chromosomes** | **Number of loci** | **New findings** |
| --- | --- | --- | --- |
| Dpfp1/Dpfp6 | 13 | 1 | Dpfp1 is constructed of 16 modules, each encoded by an exon. Fifteen of these exons encode each of the 13-residue repeats in the N-terminal half of the molecule. Dpfp6 is a splice variant consisting of 9 of these exons. |
| Dpfp2 | 6 | 1 | The N-terminal sequence was resolved, including the signal peptide. The Dpfp12 protein (from the proteome) is encoded by the N-terminal and the Dpfp2 protein by the C-terminal region of this cds, and a portion of the mid region is used in both proteins. |
| Dpfp5 | 13 | 2 | The N-terminal sequence was resolved, including the signal peptide. There are two divergent loci. |
| Dpfp7alpha | 11,12 | 3 | There is substantial coding variation between alpha, beta, gamma isoforms as the proteomics showed, but there is little or no variation between loci within these catagories. The exon/intron structure of the paralogs of alpha, beta and gamma is well conserved—possible duplications through DNA transposition. |
| Dpfp7beta | 1,4 | 2 |  |
| Dpfp7gamma | 11,12 | 3 |  |
| Dpfp8 | 3 | 2 | Dpfp8 appears to be encoded from a small portion of each of two divergent loci located 99kb apart on chromosome 3. In each locus, Dpfp8 is at the C-terminus of a much longer ORF. |
| Dpfp9alpha | 5 | 8 | There is substantial coding variation among loci. |
| Dpfp9beta | 2,5,9 | 6 |  |
| Dpfp10 | 9,13 | 5 |  |
| Dpfp11alpha | 4 | 3 |  |
