# Supplementary material for: The genome of the zebra mussel, Dreissena polymorpha: a resource for comparative genomics, invasion genetics, and biocontrol
Source: G3 (Bethesda). 2021 Dec 13;12(2):jkab423. doi: 10.1093/g3journal/jkab423 (PMC9210306; doi:10.1093/g3journal/jkab423)
Supplement: jkab423_Supplementary_Files [file jkab423_supplementary_files.zip › Supplemental info/12. Shematrin_descriptive.docx]

**Summary comparisons between *D. polymorpha* shematrin-like proteins and *Pinctada fucata* shematrins.**

A. Amino acid composition

*Dreissena polymorpha*

| **Residue** | **DPMN_136646** | **DPMN_048164** | **DPMN_126617** | **DPMN_173653** | **DPMN_173639** | **DPMN_014835** | **Mean** |
| --- | --- | --- | --- | --- | --- | --- | --- |
| Ala | 1.6 | 0.0 | 0.9 | 3.7 | 0.7 | 1.0 | 1.3 |
| Cys | 0.0 | 0.0 | 7.3 | 5.9 | 5.6 | 4.2 | 3.8 |
| Asp | 0.0 | 1.0 | 0.9 | 3.0 | 2.1 | 0.7 | 1.3 |
| Glu | 1.6 | 0.0 | 0.0 | 0.0 | 0.0 | 0.3 | 0.3 |
| Phe | 14.1 | 2.1 | 2.8 | 5.2 | 3.5 | 2.8 | 5.1 |
| Gly | 45.3 | 52.6 | 42.2 | 43.0 | 47.2 | 59.7 | 48.3 |
| His | 0.0 | 7.2 | 0.9 | 0.7 | 0.7 | 1.4 | 1.8 |
| Ile | 0.0 | 0.0 | 0.0 | 0.7 | 0.0 | 0.3 | 0.2 |
| Lys | 0.0 | 3.1 | 11.0 | 6.7 | 9.2 | 5.9 | 6.0 |
| Leu | 10.9 | 3.1 | 0.9 | 0.7 | 0.7 | 2.4 | 3.1 |
| Met | 0.0 | 0.0 | 0.9 | 0.0 | 0.7 | 0.0 | 0.3 |
| Asn | 0.0 | 0.0 | 0.0 | 0.7 | 0.0 | 0.7 | 0.2 |
| Pro | 6.3 | 3.1 | 4.6 | 1.5 | 2.8 | 0.3 | 3.1 |
| Gln | 1.6 | 0.0 | 1.8 | 1.5 | 1.4 | 1.0 | 1.2 |
| Arg | 4.7 | 2.1 | 3.7 | 4.4 | 2.8 | 1.4 | 3.2 |
| Ser | 0.0 | 1.0 | 0.9 | 1.5 | 0.7 | 1.7 | 1.0 |
| Thr | 0.0 | 1.0 | 0.9 | 0.0 | 0.7 | 0.7 | 0.6 |
| Val | 3.1 | 0.0 | 4.6 | 3.7 | 5.6 | 1.0 | 3.0 |
| Trp | 0.0 | 4.1 | 2.8 | 4.4 | 2.1 | 0.7 | 2.4 |
| Tyr | 10.9 | 19.6 | 12.8 | 12.6 | 13.4 | 13.5 | 13.8 |

*Pinctada fucata*

| **Residue** | **Shematrin1** | **Shematrin2** | **Shematrin3** | **Shematrin4** | **Shematrin5** | **Shematrin6** | **Shematrin7** | **Mean** |
| --- | --- | --- | --- | --- | --- | --- | --- | --- |
| Ala | 5.3 | 4.5 | 5.4 | 0.0 | 1.5 | 5.9 | 5.7 | 4.0 |
| Cys | 0.0 | 0.0 | 0.0 | 0.3 | 2.3 | 0.0 | 0.0 | 0.4 |
| Asp | 0.3 | 1.1 | 0.0 | 0.7 | 10.8 | 0.7 | 0.3 | 2.0 |
| Glu | 0.0 | 0.0 | 0.0 | 0.0 | 0.8 | 0.0 | 1.0 | 0.3 |
| Phe | 3.1 | 1.9 | 2.1 | 0.7 | 3.5 | 1.0 | 1.3 | 1.9 |
| Gly | 39.9 | 43.3 | 43.5 | 45.0 | 28.6 | 38.5 | 39.1 | 39.7 |
| His | 0.3 | 0.0 | 0.3 | 0.7 | 0.8 | 0.4 | 0.3 | 0.4 |
| Ile | 3.7 | 7.8 | 8.6 | 2.7 | 2.3 | 9.1 | 2.3 | 5.2 |
| Lys | 0.9 | 1.3 | 1.2 | 0.7 | 0.8 | 1.4 | 1.0 | 1.0 |
| Leu | 4.3 | 6.7 | 2.7 | 5.8 | 9.7 | 6.6 | 13.0 | 7.0 |
| Met | 0.0 | 0.0 | 0.0 | 0.0 | 1.2 | 0.0 | 2.3 | 0.5 |
| Asn | 1.5 | 1.1 | 0.6 | 2.1 | 5.8 | 2.1 | 1.3 | 2.1 |
| Pro | 4.3 | 3.7 | 3.9 | 4.8 | 1.9 | 5.9 | 5.4 | 4.3 |
| Gln | 1.2 | 1.6 | 1.5 | 0.0 | 2.3 | 0.4 | 0.3 | 1.0 |
| Arg | 1.2 | 1.9 | 1.2 | 3.4 | 11.2 | 1.0 | 4.3 | 3.5 |
| Ser | 8.7 | 8.8 | 7.1 | 7.9 | 2.7 | 7.3 | 11.0 | 7.7 |
| Thr | 2.5 | 1.9 | 3.0 | 1.4 | 0.8 | 4.2 | 0.3 | 2.0 |
| Val | 7.7 | 5.9 | 10.1 | 1.0 | 1.2 | 12.2 | 6.4 | 6.4 |
| Trp | 0.0 | 0.0 | 0.0 | 0.0 | 0.4 | 0.0 | 0.0 | 0.1 |
| Tyr | 14.9 | 8.6 | 8.9 | 22.7 | 11.6 | 3.1 | 4.3 | 10.6 |

**B. Molecular mass, pI and charge.** Molecular mass (kDa), isoelectric point (pI) and net charge values were determined using EMBOSS Pepstats.

| **Species** | **Gene ID** | **Number of Residues** | **kDa** | **pI** | **Charge** |
| --- | --- | --- | --- | --- | --- |
| *D. polymorpha* | DPMN_136646 | 64 | 6.3 | 9.33 | 2.0 |
| *D. polymorpha* | DPMN_048164 | 97 | 9.7 | 9.36 | 7.5 |
| *D. polymorpha* | DPMN_126617 | 109 | 10.9 | 9.86 | 15.5 |
| *D. polymorpha* | DPMN_173653 | 135 | 13.6 | 9.37 | 11.5 |
| *D. polymorpha* | DPMN_173639 | 142 | 13.6 | 9.62 | 14.5 |
| *D. polymorpha* | DPMN_014835 | 288 | 25.5 | 9.35 | 20.0 |
| *P. fucata* | Shematrin1 | 323 | 30.3 | 9.16 | 6.5 |
| *P. fucata* | Shematrin2 | 374 | 33.4 | 9.53 | 8.0 |
| *P. fucata* | Shematrin3 | 336 | 29.7 | 9.57 | 8.5 |
| *P. fucata* | Shematrin4 | 291 | 28.6 | 9.25 | 11.0 |
| *P. fucata* | Shematrin5 | 259 | 28.1 | 7.53 | 2.0 |
| *P. fucata* | Shematrin6 | 286 | 24.8 | 9.91 | 5.5 |
| *P. fucata* | Shematrin7 | 299 | 26.8 | 10.57 | 12.5 |
