# Supplementary material for: The genome of the zebra mussel, Dreissena polymorpha: a resource for comparative genomics, invasion genetics, and biocontrol
Source: G3 (Bethesda). 2021 Dec 13;12(2):jkab423. doi: 10.1093/g3journal/jkab423 (PMC9210306; doi:10.1093/g3journal/jkab423)
Supplement: jkab423_Supplementary_Figure_Legends_and_File_Descriptions [file jkab423_supplementary_figure_legends_and_file_descriptions.pdf]

Supplemental Figure Legends

Supplemental Figure 1. PacBio sequencing library and sequencer output.
a) Size-selected PacBio *D. polymorpha* sequencing library.
b) Distribution of subread lengths in the PacBio sequencing data set.

Supplemental Figure 2. Pulsed-field Gel Electrophoresis of input gDNA. Concentrations indicate DNA concentration at time of shearing. 200 ng DNA loaded for gel visualization in each well. Image cropped, inverted, and adjusted for brightness/contrast. Ladder 1: CHEF DNA Size Standard 8-48 kb (1703707) Ladder 2: CHEF DNA Size Standard 5 kb (1703624). White arrowhead indicates 48.5 kb.

Supplemental Figure 3. Hi-C pre- and post-scaffolding heatmaps.
a) Hi-C pre-scaffolding heatmap.
b) Hi-C post-scaffolding heatmap.

Supplemental Figure 4. Divergence trees for select orthogroups.
a) Histogram of paralog group counts for the *D. polymorpha* genome.
b-d) BEAST trees with date estimates for the expansion of several gene families in *D. polymorpha* relative to the Eastern oyster (*C. virginica*).
b) OG0000776.
c) OG0000670.
d) OG0000272.

Supplemental Figure 5. Structure of the *D. polymorpha* mitochondrial genome.
a) Dotplot (self-self) of D. polymorpha mitochondrial genome demonstrating large blocks of repetitive sequence elements. Generated in Geneious based on EMBOSS tool dottup. Word size=15, tile size = 20kb.
b) Comparison of the normalized number of Hi-C links comes from a contig within the same scaffold versus the normalized number of Hi-C links from a contig assembled on a different scaffold. The links observed between the mitochondrial contig and the chromosomal scaffolds is comparable to the number of links between contigs assembled on different scaffolds.

Supplemental Figure 6. Alignment of PacBio and Illumina reads to the mitochondrial genome.

a) Coverage plot of Minimap2-aligned PacBio reads against initial Canu-assembled mitochondrial genome (concatenated) showing area of high coverage which was determined to be a collapsed repeat sequence.

b) Paired-end Illumina reads from mixed somatic/germline male tissue were aligned to the mitochondrial genome, demonstrating a lack of SNPs that might otherwise indicate heteroplasmy. Only the coding region is shown, as unambiguous mapping of short reads to highly repetitive sequences is unreliable. Allele threshold for coverage plot = 0.05.

Supplemental Figure 7. Phylogenetic tree of the *Ty3/Gypsy* family of retrotransposons, including putative SLEs in zebra mussels.

Maximum likelihood phylogenetic tree of amino acid sequences from the entire *Gag-Pol* region. The selected model of amino acid sequence evolution was the LG (Le and Gascuel 2008) model +G (rates Gamma-distributed, α = 1.566) +I (estimated proportion of invariant sites =.001)+ F (amino-acid frequencies estimated from the data). The analysis included all sequenced elements from branch 2 of the *Ty3/Gypsy* family (including LTR retrotransposons and non-chromodomain retroviruses (Llorens *et al.* 2008), but only the Mag clades (A, B, and C in colored boxes) are shown, along with the sister clade. *Steamer* (arrow) from *Mya arenaria* groups with the sea urchin retroelement SURL, in clade C (Arriagada *et al.* 2014). Here we show that the *D. polymorpha* elements are sister to *Steamer*, confirming that they are SLEs. Bootstrap support values > 70 label the nodes, and the scale bar is expected changes per site from maximum likelihood.

Supplemental Figure 8. Partial SLEs in the *D. polymorpha* genome.

Schematic depicting incomplete SLEs, including LTR-only sequences in the *D. polymorpha* genome. Sequences are centered on an LTR element and additional annotated domains in the SLE ORF are colored as indicated in Fig. 3a.

Supplemental Figure 9. Tissue-specificity scores.

a) Histogram depicting the distribution of tissue-specificity (tau) scores for *D. polymorpha* genes. A cut-off of 0.95 was used to define tissue-specific expression (dashed line).

b) Venn diagram of tissue-specific genes compared to genes that were differentially expressed under different experimental conditions.

Supplemental Figure 10. ***D. polymorpha*** **shematrin-like proteins.**
Multiple sequence alignment of the six shematrin-like proteins identified in the *D. polymorpha* genome using CLUSTAL Omega (https://www.ebi.ac.uk/Tools/msa/clustalo/).

Supplemental Figure 11. Structures of D. polymorpha shematrin-like proteins. a-f are the six highly expressed shematrin-like proteins from mantle transcriptomes, labeled with their DPMN number. Amino acid sequences from these highly expressed genes are numbered starting from residue 1 following the signal peptide (which is not shown). Regions of low complexity (i.e. single-residue bias) detected by fLPS are labeled above the colored bars. The grey triangles below the sequences mark tandem repeat regions detected using XSTREAM. The most highly expressed gene, DPMN 014835, is shown in two segments due to its length.

Supplemental Figure 12. Structures of pearl oyster shematrin proteins. For comparison, we also examined the repetitive low complexity domains in the most well characterized shematrins, Shematrins 1-7 from *Pinctada fucata* (panels a-g). Labeling as in figure 10. Each protein is divided into two segments for clarity.

Supplemental Figure 13. Temptins and similar proteins. Yellow-highlight sequence labels are the two temptin proteins from the sea hare *Aplysia* and the two, highly expressed temptin-like proteins from zebra mussel mantle tissue, in a multiple alignment to proteins with similar domains. Sequence labels show GenBank accessions/sequence IDs followed by short names for annotated proteins (DBH = dopamine β-hydroxylase, MoxD1 = monooxygenase DBH-like 1, PHM = peptidylglycine α-hydroxylating monooxygenase). *Dreissena polymorpha* genes that show high tissue specificity are labeled with the tissue of highest expression and the τ value. The alignment starts at position 1 of the calcium-binding EGF (cbEGF)-like domain. The region near the N-terminus, including the signal peptide, is not shown. Locations of the three major domains, determined from NCBI CD searches, are labeled atop the sequences. In the cbEGF-like domain, the calcium-binding loop region is shaded in grey. Conserved residues flanking and stabilizing this region are marked (the two W residues with green arrows, and the disulfide bond between C residues with black arrows). All the DBH-like proteins contain both of the copper-binding Mox type II domains, and extend about 400 residues beyond their cbEGF-like domains. The temptins and temptin-like proteins lack the Mox domains and are much shorter, extending only about 30 residues beyond the cbEGF-like domain. The PHM-like proteins have both Mox

domains, but lack the cbEGF-like domain. And finally, all of the mollusk DBH-like proteins, and the temptin and temptin-like proteins have cbEGF-like domains containing multiple residues that are conserved among the bivalves and the gastropod *Aplysia.*

Supplemental Figure 14. Temptin and DBH-like proteins in *Aplysia*. *Aplysia californica* temptin was used in tBLASTn searches restricted to *A. californica*, and six of the top hits were aligned to temptin. The alignment produced results similar to those described above.

Supplemental Figure 15. Foot gene expression during byssal thread formation.
a) Schematic depicting experimental design; byssal threads were severed at day zero, and dissected foot tissue was collected on days zero, four, and eight (n = 4 animals per condition).
b) Gene expression changes (log2 fold-change) relative to the day zero time point.
c) List of up- and down-regulated genes in the foot at the day-four time point.

Supplemental Table 1. Sequenced bivalve genomes.

Supplemental Table 2. RepeatMasker output.

Supplemental Files

Supplemental File 1. Gene IDs for zebra mussel paralogous groups.
ZM_Paralogous_Groups.csv

Supplemental File 2. Annotation of zebra mussel paralogous groups.
ZM_GFF_Paralogous_Groups.xlsx

Supplemental File 3. Orthogroup gene IDs for zebra mussel and Eastern oyster comparison.
ZM_vs_EO_Orthogroups.txt

Supplemental File 4. Annotated orthogroups for zebra mussel and Eastern oyster comparison.
Orthogroups_EO_ZM_Products.xlsx

Supplemental File 5. Mantle-specific genes.
Mantle-specific_genes.csv

Supplemental File 6. Gill-specific genes.
Gill-specific_genes.csv

Supplemental File 7. Foot-specific genes.
Foot-specific_genes.csv

Supplemental File 8. Differentially expressed genes in mantle in mussels collected from high and low calcium environments.
Mantle_DEGs.csv

Supplemental File 9. Differentially expressed genes in the gill in mussels exposed to different thermal stress conditions.
Gill_DEGs_.csv

Supplemental File 10. Differentially expressed genes in the foot in response to severing the byssal threads.
Foot_DEGs.csv

Supplemental File 11. Mantle-specific BLAST results.
Mantle-specific_BLAST_results.xlsx

Supplemental File 12. Summary comparisons between *D. polymorpha* shematrin-like proteins and *Pinctada fucata* shematrins.
Shematrin_descriptive.docx

Supplemental File 13. Identification of full length Dpfp proteins.
Dpfp_analysis.docx

Supplemental File 14. Foot DGE BLAST results.
Foot_DEG_BLAST_results.xlsx

Supplemental File 15. Gill DGE BLAST results.
Gill_DEG_BLAST_results.xlsx

Supplemental File 16. Metadata associated with RNA-Seq samples.
Transcriptome_metadata.xlsx
